# A score-based method of immune status evaluation for healthy individuals with complete blood cell counts

**DOI:** 10.1186/s12859-023-05603-7

**Published:** 2023-12-11

**Authors:** Min Zhang, Chengkui Zhao, Qi Cheng, Jiayu Xu, Nan Xu, Lei Yu, Weixing Feng

**Affiliations:** 1 College of Intelligent Systems Science and Engineering, Harbin Engineering University, Harbin, China; 2 Institute of Biomedical Engineering and Technology, Shanghai Engineering Research Center of Molecular Therapeutics and New Drug Development, School of Chemistry and Molecular Engineering, East China Normal University, Shanghai, China; 3 Shanghai Unicar-Therapy Bio-Medicine Technology Co., Ltd, Shanghai, China

**Keywords:** Immune status evaluation, Healthy individuals, Complete blood counts, Clustering, Sub-healthy

## Abstract

**Background:**

With the COVID-19 outbreak, an increasing number of individuals are concerned about their health, particularly their immune status. However, as of now, there is no available algorithm that effectively assesses the immune status of normal, healthy individuals. In response to this, a new score-based method is proposed that utilizes complete blood cell counts (CBC) to provide early warning of disease risks, such as COVID-19.

**Methods:**

First, data on immune-related CBC measurements from 16,715 healthy individuals were collected. Then, a three-platform model was developed to normalize the data, and a Gaussian mixture model was optimized with expectation maximization (EM-GMM) to cluster the immune status of healthy individuals. Based on the results, Random Forest (RF), Light Gradient Boosting Machine (LightGBM) and Extreme Gradient Boosting (XGBoost) were used to determine the correlation of each CBC index with the immune status. Consequently, a weighted sum model was constructed to calculate a continuous immunity score, enabling the evaluation of immune status.

**Results:**

The results demonstrated a significant negative correlation between the immunity score and the age of healthy individuals, thereby validating the effectiveness of the proposed method. In addition, a nonlinear polynomial regression model was developed to depict this trend. By comparing an individual’s immune status with the reference value corresponding to their age, their immune status can be evaluated.

**Conclusion:**

In summary, this study has established a novel model for evaluating the immune status of healthy individuals, providing a good approach for early detection of abnormal immune status in healthy individuals. It is helpful in early warning of the risk of infectious diseases and of significant importance.

**Supplementary Information:**

The online version contains supplementary material available at 10.1186/s12859-023-05603-7.

## Background

It is quite challenging to detect diseases in their early stages. When symptoms appear, the immune system’s balance may be severely disrupted, and its defense ability is greatly weakened [1, 2]. Therefore, it is of great significance to identify the abnormal immune status in healthy individuals as early as possible. This can contribute to providing early warnings about the risk of contracting diseases, such as COVID-19.

Walford proposed the theory of immunosenescence, which suggests that the decline in immune functions is the primary factor for aging [3–6]. This decline involves structural and functional changes in immune organs, particularly a decrease in the capability of immune cells. It weakens the body’s ability to resist infections and leads to a higher prevalence of autoimmune diseases, chronic inflammation, and even cancers [7]. However, aging affects individuals to different extents. A recent study has shown that the immune status of healthy individuals is continuous rather than discrete, meaning that individuals of the same age may differ in their physiological age in terms of immunity. This high inter-individual variability emphasizes the need for quantitative evaluation of immune status to study the gradual changes occurring in the immune system [5, 8–18]. Currently, clinicians generally assess the immune status of patients based on the presence of basic diseases such as diabetes, malignant tumors, and chronic renal failure. However, this approach lacks precision [19]. Immunologists are seeking ways to directly assess the immune status of humans to improve our understanding of the human immune system [20–23]. Typical methods for assessing immune function include clinical evaluation of susceptibility to infections. For example, the Jeffrey Modell Foundation (JMF) has developed a set of warning signs for 10 primary immunodeficiency diseases that may indicate susceptibility to infection [24]. Analyzing a combination of autoimmune, allergic, or malignant tumor immunodeficiency states is another assessment method. Formal laboratory evaluation of immune status can be performed in multiple ways, such as measuring immunoglobulins, IgG subclasses, complement function, counts of T cells, B cells, NK cells, vaccine responses, and T cell proliferation. Deviations in these indicators from the reference range can have clinical significance in assessing immune functions and diagnosing diseases. However, these methods primarily focus on populations that already have immune problems and are not sensitive enough to detect subtle immune changes in healthy individuals. Although these methods have certain clinical value in the diagnosis and treatment of certain diseases, they are all aimed at populations that already have immune problems, and are not sensitive enough to detect subtle immune changes in healthy individuals.

Some researchers have attempted to evaluate human immune states based on the number and function of lymphocytes [19]. However, while these studies compared individuals of different ages, they did not develop an immune state assessment model. Another study attempted to establish an immune scoring model based on the combination of lymphocyte number, function, and phenotype [25]. Although this model enabled the assessment of an individual’s immune status, it was relatively simplistic and only indicated whether the results fell within the normal range or exceeded the limit. To date, few studies have focused on developing an algorithm that provides continuous immunity scores for healthy individuals to assess their immune status.

In this study, we collected complete blood cell counts from a large cohort of healthy individuals. We measured the absolute counts and percentages of various immune cell types, including white blood cells, lymphocytes, neutrophils, eosinophils, and basophils, along with their corresponding percentages. Using these measurements, we developed an immune status assessment model. We then investigated the relationship between the immunity score generated by the model, age, and lifestyle factors such as staying up late. Our findings suggest that age and lifestyle factors have a significant impact on immune status in healthy individuals, and our model effectively measures this impact. Developing a simple, reliable, and inexpensive method to evaluate immune status is crucial for enhancing our understanding of immune function and promoting better health. With our method, healthy individuals can easily monitor their immune status and identify early changes that may lead to immune-related diseases.

## Methods

**Fig. 1:**
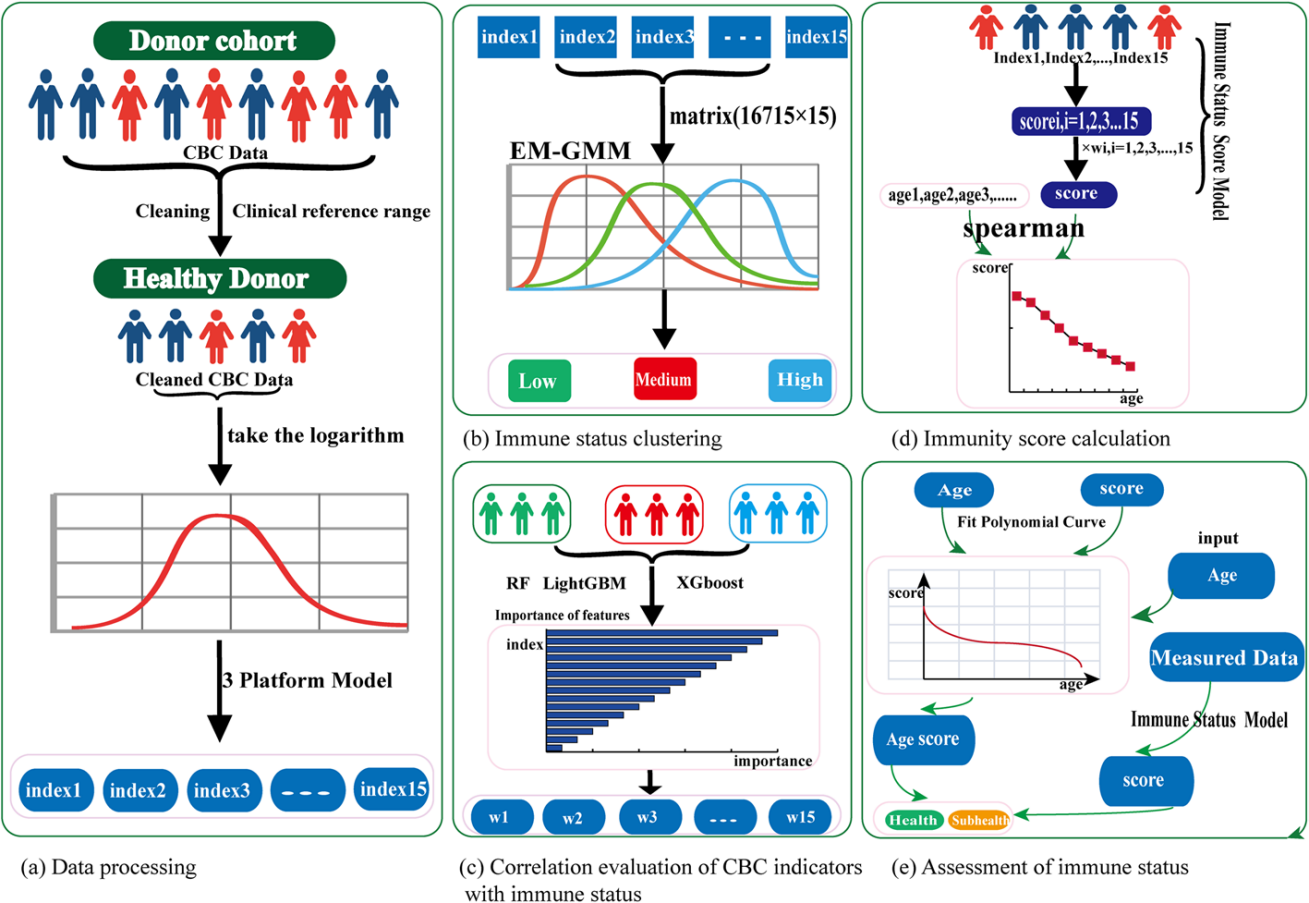
Overall workflow of the study. **a** Data processing. **b** Immune status clustering. **c** Correlation evaluation of CBC indexes with immune status. **d** Immunity score calculation. **e** Assessment of immune status

Currently, there is a lack of methods that comprehensively assess the immune status of healthy individuals. To address this gap, this study developed a machine learning-based approach to enhance the evaluation of immune status using complete blood count (CBC) data. The study was conducted in five distinct stages: data processing, immune status clustering, correlation evaluation of CBC indexes with immune status, score calculation of immunity, and immune status assessment. First, the CBC data obtained from the physical examination of healthy individuals underwent a cleaning process based on inflammatory indexes, as shown in Fig. 1a. Then, a three-platform model was devised to normalize the data, and an optimization of the Gaussian mixture model using the expectation–maximization (EM-GMM) technique was performed to cluster the immune status of the healthy participants, as shown in Fig. 1b. Using the obtained results, RF, LightGBM and XGBoost algorithms were employed to assess the correlation between each CBC index and immune status, as shown in Fig. 1c. Based on these findings, an assessment model was constructed to calculate a continuous immunity score for healthy individuals, as shown in Fig. 1d. Lastly, a nonlinear polynomial regression model was developed to further evaluate an individual's immune status, as shown in Fig. 1e. The overall workflow of the study is illustrated in Fig. 1.

## Data

### Data acquisition

The study involved the collection of complete blood cell counts (CBC) data from a total of 19,102 adults aged 20–84 years. Among the participants, there were 11,041 (57.8%) males and 8061 (42.2%) females. To ensure data accuracy, certain measures were taken to remove any potential interference. Firstly, data with procalcitonin (PCT) levels exceeding 0.5 ng/mL, which is often indicative of bacterial infection, were excluded from the analysis [26, 27]. Subsequently, data points that fell outside the clinical normal reference range for inflammatory-related indicators were also eliminated. The retained dataset consisted of CBC data from healthy individuals, with white blood cell counts ranging from 4 to 10 (10^9^/L), neutrophil counts ranging from 2 to 7 (10^9^/L), lymphocyte counts ranging from 0.8 to 4 (10^9^/L), neutrophil percentages ranging from 40 to 75%, and lymphocyte percentages ranging from 20 to 50%. Ultimately, immune-related CBC data from 16,715 healthy individuals were obtained, comprising 9831 (58.8%) males and 6884 (41.2%) females.

The study considered a total of 15 immune-related CBC indexes, including white blood cell count (WBC), lymphocyte count (LYMPH), neutrophil count (NEUT), monocyte count (MONO), eosinophil count (EO), basophil count (BASO), lymphocyte percentage (LYMPH%), neutrophil percentage (NEUT%), monocyte percentage (MONO%), eosinophil percentage (EO%), basophil percentage (BASO%), neutrophil to lymphocyte ratio (NLR), monocyte to lymphocyte ratio (MLR), eosinophil to lymphocyte ratio (ELR), and basophils to lymphocytes ratio (BLR).

To analyze the data, each index was logarithmized, and their distributions were confirmed to be Gaussian, as depicted in Additional file 1: Fig. S1A. The mean value (μ) and standard deviation (σ) for each index were calculated and presented in Additional file 2: Table S1. A comparison of different indexes within the same coordinate system is displayed in Additional file 1: Fig. S1B.

Based on previous studies, it is well-established that the human immune status tends to decline with age [25, 28–31]. To investigate the changes in each CBC index, Spearman correlation tests were conducted between each index and age. The results indicated significant correlations between age and all indexes except NEUT. Notably, WBC, LYMPH, and LYMPH% exhibited negative correlations with age. These correlation results are presented in Additional file 1: Fig. S1C.

### Data normalization

To address the magnitude bias among different indexes (Additional file 1: Fig. S1B), all data were normalized to a range of 0 to 1. Taking into account the biological characteristics, a normalized function was designed to simulate an S-shaped growth curve, which was defined as a three-platform model. The two platforms at the maximum and minimum values represent the saturation states of immune status, while an additional platform near the median value reflects the self-regulation ability of the human immune system.

Spearman correlation analysis was performed separately for each CBC index and age, considering different genders. The results indicated consistent directions of the relationship between CBC indices and age for both males and females. Additional file 1: Fig. S2 provides a visual representation of these correlation results. Consequently, data from different genders were combined for further analysis in this study.

Based on the correlation between each index and age (Additional file 1: Fig. S3), it was observed that the quantities of WBC, LYMPH, NEUT, and LYMPH% tended to decrease with age. The three-platform models for these four indicators can be represented by Eq. (1):

1 $$\tilde{x} = \left\{ {\begin{array}{*{20}c} {\frac{1}{2}e^{{\tfrac{{ - (x - \mu )^{2} }}{{2\sigma^{2} }}}} } & {(x \le \mu )} \\ {1 - \frac{1}{2}e^{{\tfrac{{ - (x - \mu )^{2} }}{{2\sigma^{2} }}}} } & {(x > \mu )} \\ \end{array} } \right.$$

where μ denotes the mean value and σ indicates the standard deviation of each index after logarithmization.

In contrast, the quantities of MONO, EO, BASO, NEUT%, MONO%, EO%, BASO%, NLR, MLR, ELR, and BLR tended to increase with age. The Three-platform models for these 11 indicators can be expressed by Eq. (2):

2 $$\tilde{x} = \left\{ {\begin{array}{*{20}c} {1 - \frac{1}{2}e^{{\tfrac{{ - (x - \mu )^{2} }}{{2\sigma^{2} }}}} } & {(x \le \mu )} \\ {\frac{1}{2}e^{{\tfrac{{ - (x - \mu )^{2} }}{{2\sigma^{2} }}}} } & {(x > \mu )} \\ \end{array} } \right.$$

These two functions have similar forms, and both including two Gaussian functions. The main difference lies in that Eq. (1) takes one form when x is less than or equal to a mean value $\mu$, and another form when x is greater than $\mu$; whereas Eq. (2) does the opposite, taking the second form of Eq. (1) when x is less than or equal to $\mu$ and the first form when x is greater than $\mu$.

### Clustering analysis using EM-GMM algorithm

In this study, we employed two commonly used unsupervised clustering methods, namely the Expectation–Maximization Gaussian Mixture Model (EM-GMM) algorithm and the k-means algorithm. To determine the most suitable clustering method for the data and the appropriate number of clusters (K value), we conducted a comprehensive evaluation using key metrics such as silhouette coefficient, Calinski–Harabasz index, and Davies–Bouldin index.

According to the Silhouette Coefficient, a measure of clustering effectiveness, which considers both the cohesion and separation of samples after clustering, a higher value closer to 1 indicates a better clustering result, where samples within the same cluster are closer and samples from different clusters are farther. Conversely, a value closer to − 1 indicates a poorer clustering result. Equation (3) shows the calculation of silhouette coefficient:

3 $$s\left( i \right) = \frac{b\left( i \right) - a\left( i \right)}{{\max \left( {a\left( i \right),b\left( i \right)} \right)}}$$

where $a(i)$ measures the similarity within the group, $b(i)$ measures the similarity between groups, and $s(i)$ ranges from − 1 to 1, with higher values indicating higher similarity within groups and greater distance between groups.

According to the Calinski–Harabasz index, a metric for evaluating clustering quality, a higher value signifies better clustering results. It considers both the within-cluster dispersion and the between-cluster dispersion. The index is calculated using Eq. (4).

4 $$CH = \frac{{B_{k} }}{{W_{k} }} \times \frac{N - k}{{k - 1}}\;$$

where $B_{k}$ represents the between-cluster dispersion, $W_{k}$ represents the within-cluster dispersion, N is the total number of samples, and k is the number of clusters. A higher index value indicates a stronger separation between clusters and a more compact cluster structure.

On the other hand, the Davies–Bouldin index measures the average similarity between clusters and takes into account both the within-cluster scatter and the between-cluster separation. To calculate the index, we employ Eq. (5).

5 $$DB = \frac{1}{k}\sum\limits_{i = 1}^{k} {\max_{j \ne i} R_{ij} }$$

where $R_{ij}$ is the similarity measure between clusters $C_{i}$ and $C_{j}$. A lower Davies-Bouldin index indicates better clustering results, with well-separated and distinct clusters having smaller values. The evaluation results of the clustering performance metrics for the two unsupervised clustering methods under different numbers of clusters are presented in Table 1.

**Table 1:** Comparison of EM-GMM and K-means clustering performance

Clustering algorithm	Silhouette coefficient	Calinski–Harabasz index	Davies–Bouldin index
EM-GMM (K = 2)	0.391	10,447.381	1.178
EM-GMM (K = 3)	**0.400**	11,285.403	**1.073**
EM-GMM (K = 4)	0.398	**12,465.243**	1.235
K-means (K = 2)	0.183	4015.301	1.921
K-means (K = 3)	0.179	3550.229	1.710
K-means (K = 4)	0.145	3165.865	1.733

The bold font indicates the optimal value for this parameter

Therefore, taking into account the silhouette coefficient, Calinski-Harabasz index, Davies–Bouldin index, and the biological significance, we utilized the EM-GMM algorithm to cluster the immune states into three categories.

Gaussian mixture model(GMM) is an unsupervised learning model, which is a linear combination of multiple nonlinear Gaussian distribution functions. In theory, GMM can fit any type of distribution, and is usually used to simulate data containing different distributions with the same type but different parameters. The input here was a matrix of 16,715 × 15 dimensions, and the expectation–maximization Gaussian mixture algorithm (EM-GMM) was adopted to determine features and perform unsupervised clustering simultaneously. The 15 indicators used for clustering were described in the subsection “Data acquisition”.

The number of clusters K was set to 3 trying to divide human immune status into three categories: high, medium, and low. That means the Gaussian mixture model should consist of 3 Gaussian “Component”, and these components are linearly superimposed together to form the probability density function of the GMM, which is depicted as Eq. (6):

6 $$p\left( x \right) = \sum\limits_{k = 1}^{K} {p\left( k \right)p\left( {x|k} \right) = } \sum\limits_{k = 1}^{K} {\pi_{k} {\rm N}\left( {x{|}\mu_{k} ,\Sigma_{k} } \right)}$$

where $\mu_{k}$ is the mean value, $\Sigma_{k}$ is the covariance matrix,$p\left( {x|k} \right){ = }{\rm N}\left( {x{|}\mu_{k} ,\Sigma_{k} } \right)$ is called conditional probability of the *k*th component in the mixture model,$p\left( k \right){ = }\pi_{k}$ refers to the probability when component *K* is selected, and satisfies $\Sigma_{k = 1}^{K} \pi_{k} { = }1\left( {0 \le \pi_{k} \le 1} \right)$.

A 3-dimensional latent variable $z$ is introduced with the value of 0 or 1, and $z_{k} { = 1}$ means the sample is selected. The posterior probability indicates the possibility of the *i*th data belonging to the category *k*, which satisfies $\Sigma_{k = 1}^{K} \gamma \left( {z_{ik} } \right) = 1$ and $\gamma \left( {z_{ik} } \right) \in \left\{ {0,1} \right\}$, as shown in Eq. (7):

7 $$\gamma \left( {z_{ik} } \right){\text{ = p}}\left( {x_{i} ,z;\theta } \right) = \frac{{p\left( {x,z_{k} = 1} \right)}}{p\left( x \right)} = \frac{{\pi_{k} {\rm N}\left( {x_{i} {|}\mu_{k} ,\Sigma_{k} } \right)}}{{\Sigma_{j = 1}^{K} \pi_{j} {\rm N}\left( {x_{i} {|}\mu_{j} ,\Sigma_{j} } \right)}}$$

Assuming that there are *N* samples, and each of them follows a certain type of distributions $p\left( x \right)$. Then the parameters $\theta { = }\left( {\pi_{k} ,\mu_{k} ,\Sigma_{k} } \right)$ need to be determined to maximize the probability of observing these samples from the mixture distributions. The log-likelihood form is in Eq. (8):

8 $$l\left( \theta \right){ = }\sum\limits_{i = 1}^{N} {\ln {\text{p}}\left( {x_{i} ,z;\theta } \right)} = \sum\limits_{i = 1}^{N} {\ln \left[ {\sum\limits_{k = 1}^{K} {\pi_{k} {\rm N}\left( {x_{i} |\mu_{k} ,\Sigma_{k} } \right)} } \right]}$$

Then, we apply the derivation to solve the maximum likelihood problem:

9 $$\frac{\partial l\left( \theta \right)}{{\partial \mu_{k} }} = - \sum\limits_{i = 1}^{N} {\frac{{\pi_{k} {\rm N}\left( {x_{i} {|}\mu_{k} ,\Sigma_{k} } \right)}}{{\Sigma_{j = 1}^{K} \pi_{j} {\rm N}\left( {x_{i} {|}\mu_{j} ,\Sigma_{j} } \right)}}\Sigma_{k}^{ - 1} \left( {x_{i} - \mu_{k} } \right)}$$

10 $$\frac{\partial l\left( \theta \right)}{{\partial \Sigma_{k} }} = \sum\limits_{i = 1}^{N} {\frac{{\pi_{k} {\rm N}\left( {x_{i} {|}\mu_{k} ,\Sigma_{k} } \right)}}{{\Sigma_{j = 1}^{K} \pi_{j} {\rm N}\left( {x_{i} {|}\mu_{j} ,\Sigma_{j} } \right)}}} \left( { - \frac{1}{2}\Sigma_{k}^{ - 1} + \frac{1}{2}\Sigma_{k}^{ - 1} \left( {x_{i} - \mu_{k} } \right)\left( {x_{i} - \mu_{k} } \right)^{T} \Sigma_{k}^{ - 1} } \right)$$

We let the derivatives equal to 0 and get the optimal values of mean $\mu_{k}$ and variance $\Sigma_{k}$:

11 $$\mu_{k} = \frac{1}{{N_{k} }}\sum\limits_{i = 1}^{N} {\gamma \left( {z_{ik} } \right)x_{i} }$$

12 $$\Sigma_{k} = \frac{1}{{N_{k} }}\sum\limits_{i = 1}^{N} {\gamma \left( {z_{ik} } \right)\left( {x_{i} { - }\mu_{k} } \right)\left( {x_{i} { - }\mu_{k} } \right)^{T} }$$

where $N_{K} = \Sigma_{i = 1}^{N} \gamma \left( {z_{ik} } \right)$ represents the number of samples belonging to the *k*th component of the model.

Next, in order to find the component probability $\pi_{k}$, it is necessary to use the Lagrangian operator $\Sigma_{k = 1}^{K} \pi_{k} { = }1$.

13 $$l\left( {\theta^{\prime } } \right) = l\left( \theta \right) + \lambda \left( {\Sigma_{k = 1}^{K} \pi_{k} { - }1} \right)$$

The derivative of $\pi_{k}$ is defined in Eq. (14) and the value can be obtained in Eq. (15):

14 $$\frac{{\partial l\left( {\theta ^{\prime } } \right)}}{{\partial \pi _{k} }} = \sum\limits_{{i = 1}}^{N} {\frac{{{\text{N}}\left( {x_{i} |\mu _{k} ,\Sigma _{k} } \right)}}{{\Sigma _{{j = 1}}^{K} \pi _{j} {\text{N}}\left( {x_{i} |\mu _{j} ,\Sigma _{j} } \right)}}} + \lambda = 0$$

15 $$\pi_{k} = \frac{{N_{k} }}{N}$$

The steps of the EM algorithm are listed in Table 2. It is necessary to use the algorithm to find a set of parameter values to maximize the Eq. (8) until convergence. In this study, the maximum number of iterations times was set to 100.

**Table 2:** Steps of EM algorithm

EM algorithm:
*Step*1:
The number of categories *K* is preset as 3, then set the initial values of $\theta$ for each component *K* and calculate the log-likelihood value in Eq. (7)
*Step*2: E step
Based on current values of $\theta$, the value of z for each sample is estimated
*Step*3:M step
The values of z in Eq. (7) are updated, and the log-likelihood value is maximized to get a new set of $\theta$ values
*Step4*:
Return to Step 2 until convergence

### Correlation evaluation of CBC indexes with immune status

In this section, correlation was evaluated between each CBC index and the immune status of healthy individuals. Random forest, LightGBM (Light Gradient Boosting Machine), and XGBoost (Extreme Gradient Boosting) are three widely used machine learning algorithms, all of which are adept at ranking the effect of input factors during classification [32].

Random forest (RF) is an algorithm that integrates multiple decision trees through ensemble learning, so that it has better generalization ability [33]. For a certain input sample, each decision tree is a classifier, and *N* trees will get *N* classification results. The RF can integrate all classification results through taking the most voted class as the final output. The dataset was split into a training set accounting for 80% of all and a test set of 20%. The model parameters were set as n_estimators = 100, random_state = 1, n_jobs = − 1. The RF classification algorithm is shown in Additional file 1: Fig. S4.

LightGBM is another framework that implements the GBDT algorithm, which supports efficient parallel training, and has faster training speed, lower memory consumption and better accuracy [34]. This method has been applied to the interpretability of classification, as evidenced by previous studies [35]. The dataset was also split into a training set accounting for 80% of all and a test set of 20%, and tenfold cross-validation was used to adjust hyperparameters to build the best model [36]. The model parameters were set as num_leaves = 31, learning_rate = 0.1, n_estimators = 40, max_bin = 256, max_depth = − 1. The LightGBM classification algorithm is presented in Additional file 1: Fig. S5.

XGBoost is another widely used machine learning algorithm that performs exceptionally well in various classification tasks. Standing for Extreme Gradient Boosting, XGBoost is an optimized distributed gradient boosting library designed to be efficient, flexible, and portable. Similar to RF and LightGBM, the dataset was divided into 80% for training and 20% for testing. Hyperparameters were optimized through tenfold cross-validation to build the best model, with model parameters set as max_depth = 3, learning_rate = 0.1, n_estimators = 100.

After classification through RF, LightGBM and XGBoost algorithms, the effect of each CBC indexes could be evaluated, which would be used to reflect the correlation degree of each CBC index with human immune status.

### Immunity score calculation

Altogether, *N* experiments were conducted (*N* was set to 200), where the training set and test set in each experiment changed randomly. The mean value of the correlations between each index and human immune status was adopted as the weight of the index $w_{i}$, Eventually, the weighted sum of indexes was calculated as the individual's immunity score, which is shown in Eq. (16):

16 $$score = \sum\limits_{i}^{15} {w_{i} score_{i} } \quad (i = 1,2, \ldots ,15)$$

where *score*_*i*_ represents the score of the *i*th of 15 indexes, which was calculated with the designed three-platform model.

### Nonlinear polynomial regression model for assessing immune status with age

In this section, we aimed to determine the appropriate order of polynomial regression to evaluate an individual’s immune status score with age. We considered linear, quadratic, cubic, and quartic polynomial regression models. To assess the performance of each model, we compared their mean squared error (MSE) values. However, when selecting the model complexity, it is important to consider not only the MSE but also the model’s generalization ability.

We plotted the MSE values for different polynomial orders in Additional file 1: Fig. S6. It can be observed that the fourth-degree polynomial regression model has the lowest MSE value, indicating a better fit to the data. Additionally, the third-degree polynomial regression model exhibits a slightly higher MSE compared to the fourth-degree model. We opted for the third-degree polynomial regression model based on its balance between model complexity and generalization ability. Although non-linear regression approaches may be challenging to interpret compared to linear regression, they can effectively capture the nonlinearity between an individual's immunity score and age.

In conclusion, we selected the third-degree polynomial regression model for evaluating immune status due to its reasonable fit to the data and its ability to capture the nonlinearity in the relationship between an individual's immunity score and age.

The model here is a univariate cubic polynomial regression model, where *x* means age and *f* (x) means the normal immunity score of the age. The formula is as follows:

17 $$f\left( x \right) = a_{3} x^{3} + a_{2} x^{2} + a_{1} x + a_{0}$$

If an individual’s immunity score is higher than the normal immunity score of his age, his immune status is healthy, on the contrary, his immune status is sub-healthy, so as to describe the immune status of each individual more accurately.

## Results

### Data processing

The participants in this study were adults aged 20–84. Previous research has highlighted that chronic inflammation can be a common underlying cause of various diseases. Even in the absence of apparent injury or disease, a low level of inflammation can be activated. In such cases, the immune system triggers white blood cells to attack nearby healthy tissues and organs, initiating a chronic inflammatory process. This process plays a central role in the development of challenging diseases such as rheumatoid arthritis, cancer, heart disease, diabetes, asthma, and even Alzheimer's disease [37, 38]. Therefore, in order to construct an accurate immune status evaluation model for healthy individuals, it was necessary to clean the CBC data by removing individuals with mild inflammation. Subsequently, the remaining data could more precisely represent the immune status of healthy individuals.

**Fig. 2:**
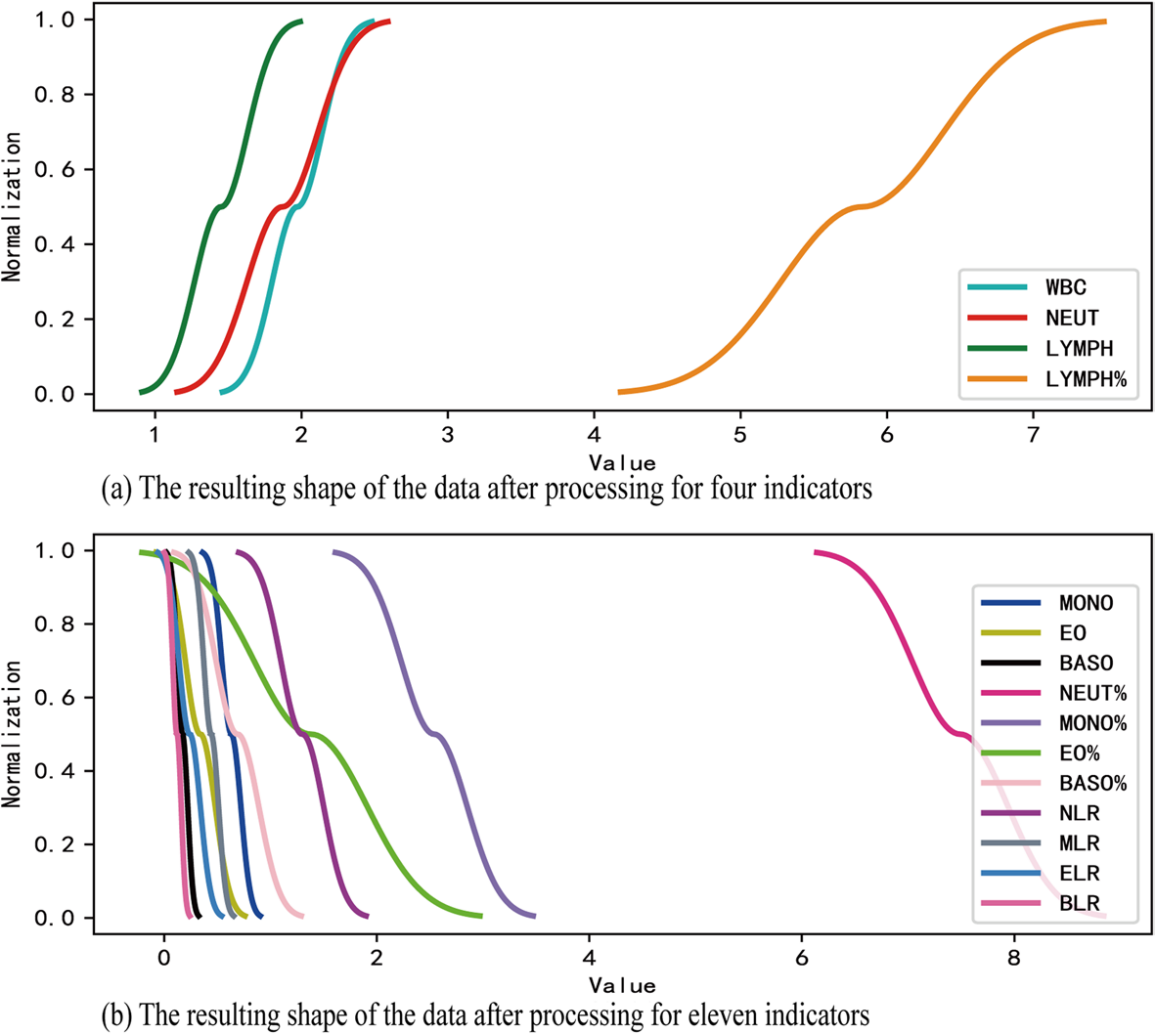
Three-platform model for normalization of CBC data. **a** The resulting shape of the data after processing for four indicators. **b** The resulting shape of the data after processing for eleven indicators

After the data cleaning process, the CBC data of 16,715 healthy individuals were normalized using the three-platform model. The resulting shape of the data after processing is depicted in Fig. 2.

### Clustering of immune status

**Fig. 3:**
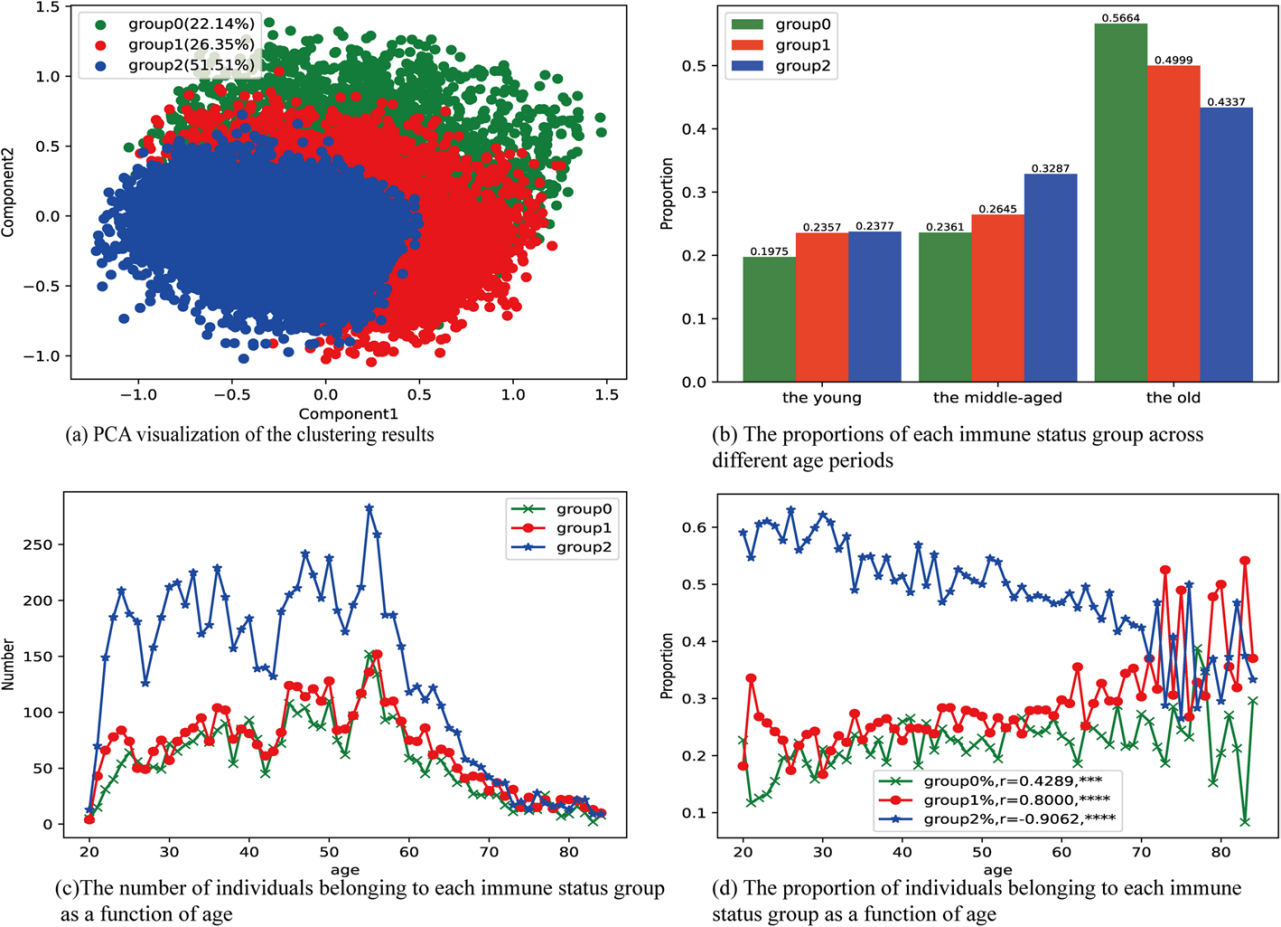
The clustering results of immune status for healthy individuals. **a** PCA visualization of the clustering results; **b** The proportions of each immune status group across different age periods (the young: 20–40; the middle-aged: 41–60; the old: > 60); **c** The number of individuals belonging to each immune status group as a function of age; **d** The proportion of individuals belonging to each immune status group as a function of age (*P < 0.05, **P < 0.01, ***P < 0.001, ****P < 0.0001)

In this study, the EM-GMM algorithm was employed to cluster the immune status of healthy individuals. The results of the clustering analysis are presented in Fig. 3. Among the three groups, Group 0 had the highest proportion among the elderly individuals and the lowest proportion among the young individuals (Fig. 3b). Furthermore, the proportion of Group 0 showed a positive correlation with age (Fig. 3d; r = 0.4289; ***, Spearman’s correlation). Thus, Group 0 was classified as the poor immune status group. On the other hand, Group 2 exhibited the lowest proportion among the elderly individuals and the highest proportion among the young individuals. Moreover, the proportion of Group 2 displayed a negative correlation with age (Fig. 3d; r = − 0.9062; ****, Spearman’s correlation). Therefore, Group 2 was identified as the good immune status group. Group 1, situated between the other two groups, was designated as the medium immune status group (Fig. 3d; r = 0.80000; ****, Spearman’s correlation).

### Correlations between CBC indexes and immune status

The correlation between each CBC index and the immune status of healthy individuals was evaluated using RF, LightGBM, and XGBoost. The data was divided into a training set (80%) and a test set (20%), with three labels representing immune status categories: poor, medium, and good. The test results of the three models are presented in Table 3. The confusion matrix and ROC for RF, LightGBM, and XGBoost are presented in Additional file 1: Figs. S7, S8 and S9, respectively.

**Table 3:** Test results of the three integrated learning models

Model	Group	Precision	Recall	F1-score	Accuracy	AUC
RF	Group0	0.97	0.98	0.97	0.977	1
	Group 1	0.96	0.97	0.97		1
	Group 2	0.99	0.98	0.98		1
LightGBM	Group 0	0.98	0.99	0.98	0.982	1
	Group 1	0.98	0.96	0.97		1
	Group 2	0.99	0.99	0.99		1
XGBoost	Group0	0.99	0.97	0.98	0.979	1
	Group 1	0.96	0.97	0.96		1
	Group 2	0.99	0.99	0.99		1

Finally, the RF, LightGBM and XGBoost models were validated using a tenfold cross-validation approach, demonstrating similar accuracy results for all methods. The results are presented in Additional file 1: Fig. S10. Therefore, to comprehensively consider both approaches, the test results of the trained three models were averaged to assess the correlation between each CBC index and the immune status of healthy individuals. The results are summarized in Table 4.

**Table 4:** The correlation degree between each CBC index and the immune status of healthy individuals

Parameter	RF	LightGBM	XGBoost	Mean
WBC	0.018	0.049	0.066	0.044
NEUT	0.015	0.041	0.049	0.035
LYMPH	0.030	0.149	0.126	0.101
MONO	0.011	0.035	0.037	0.028
EO	0.162	0.102	0.069	0.111
BASO	0.081	0.042	0.038	0.053
NEUT (%)	0.041	0.031	0.038	0.037
LYMPH (%)	0.101	0.084	0.079	0.088
MONO (%)	0.012	0.045	0.046	0.035
EO (%)	0.119	0.027	0.029	0.059
BASO (%)	0.056	0.039	0.036	0.044
NLR	0.073	0.042	0.058	0.058
MLR	0.044	0.088	0.089	0.074
ELR	0.135	0.115	0.119	0.123
BLR	0.102	0.111	0.122	0.111

### Internal validation of immune status assessment model

**Fig. 4:**
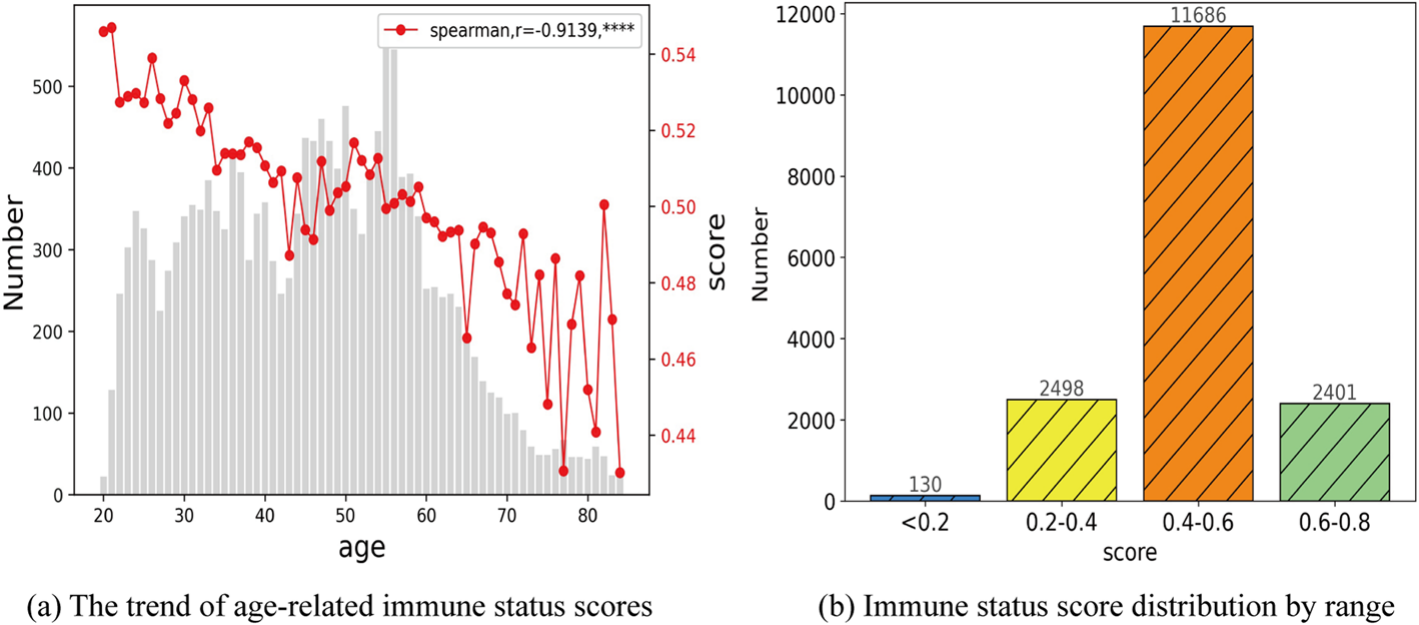
The relationship between immune status scores and age. **a** The trend of age-related immune status scores. **b** Immune status score distribution by range

The designed three-platform model was applied to normalize the values of each CBC index in healthy individuals. Subsequently, the weighted sum of the normalized values was computed as the final score for assessing immune status. The relationship between the immune status scores and the ages of the 16,715 healthy samples was analyzed and is depicted in Fig. 4.

As shown in Fig. 4a, the median values of immune status scores exhibited a decreasing trend and were found to be significantly correlated with age (r = − 0.9139, Spearman correlation coefficient), indicating a consistent pattern with the observed data. In Fig. 4b, the distribution of individuals based on different ranges of immune status scores is presented. The numbers of individuals falling into the ranges of 0.6–0.8, 0.4–0.6, 0.2–0.4, and < 0.2 were 2401 (14.4%), 11,686 (69.91%), 2498 (14.94%), and 130 (0.78%), respectively. This distribution follows a reasonable Gaussian pattern, with the average value being close to 0.5.

### Evaluating immune status with polynomial regression model

**Fig. 5:**
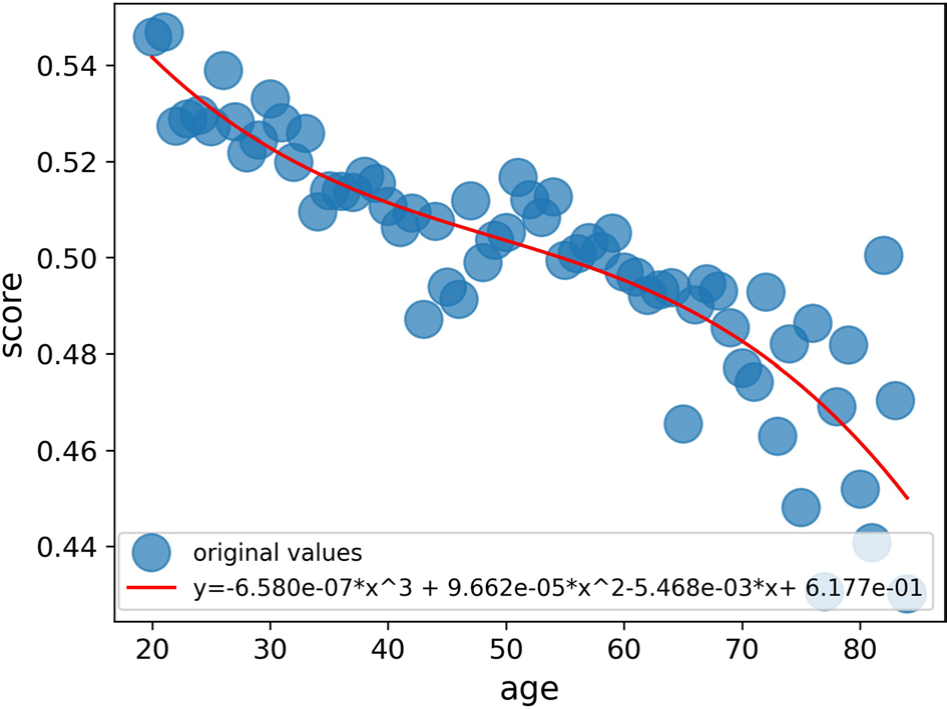
Fitted values of the immunity scores as a function of age

The immune status reflects the overall vitality and health of an individual. In this study, a cubic polynomial regression model was utilized to capture the relationship between immunity scores and age, allowing us to visualize the trend of immune status across different ages. The fitted curve, as shown in Fig. 5, provides valuable insights into interpreting an individual's immune status. Based on the curve, if a person’s immunity score exceeds the fitted value for their age, it indicates a healthy immune status. Conversely, if their immunity score falls below the fitted value, it suggests a suboptimal or sub-healthy immune status. This representation vividly portrays the immune status of an individual and facilitates a comprehensive understanding of their overall health.

### External validation of immune status evaluation model

#### Validation of immune status model using longitudinal data in healthy individuals

The immune status of five healthy individuals was monitored using the proposed immune status evaluation model for a period of ten days. The results of the monitoring are presented in Table 5. Each person underwent five consecutive CBC tests using the same instrument, with tests conducted every two days. During the monitoring period, the individuals deliberately stayed up late on specific days to intentionally disrupt their immune status. The interference days for each person were as follows: Person 1 (Day 5), Person 2 (Days 3 and 7), Person 3 (Days 5 and 9), Person 4 (Days 1, 5, 7, and 9), and Person 5 (Days 3, 5, and 7). The raw data obtained from these tests can be found in Additional file 2: Tables S2–S6.

**Table 5:** Monitoring of immune status based on the immunity scores

Date	Person1 (27 years, female)	Person2 (23 years, male)	Person3 (26 years, female)	Person4 (29 years, male)	Person5 (23 year, male)
Day 1	0.604	0.581	0.624	**0.462**	0.522
Day 3	0.621	**0.532**	0.600	0.521	**0.428**
Day 5	**0.530**	0.599	**0.579**	**0.423**	**0.456**
Day 7	0.645	**0.550**	0.657	**0.418**	**0.463**
Day 9	0.601	0.550	**0.574**	**0.341**	0.499
Ref	0.528	0.535	0.529	0.524	0.535

The immunity scores presented in bold font represent the immune status scores obtained under sleep deprivation conditions. Ref: reference immunity score

Among the five individuals, the immunity scores remained relatively stable throughout the monitoring period. The immunity scores of persons 1, 2, and 3 exceeded the reference immunity score, indicating that their immune status was healthy. On the other hand, persons 4 and 5 regularly stayed up late, and their immunity scores were lower than the reference immunity score, suggesting that their immune status was sub-healthy. Furthermore, it is worth noting that the immunity scores of person 1 on Day 5, person 2 on Days 3 and 7, person 3 on Days 5 and 9, person 4 on Days 1, 5, 7, and 9, and person 5 on Days 3, 5, and 7 all showed a slight decrease. This decrease may be attributed to insufficient sleep caused by staying up late. It is evident that staying up late and experiencing insufficient sleep can have an impact on the individual's immune status.

#### External validation in diverse healthy individuals

In this study, we conducted independent validation of our model using a separate dataset. The validation dataset consisted of CBC data from 40 healthy individuals, collected from a different device, a different batch, and a separate group of subjects as compared to the data used for model establishment. We applied the method described in this article to calculate the immune status score for each individual in the validation dataset.

**Fig. 6:**
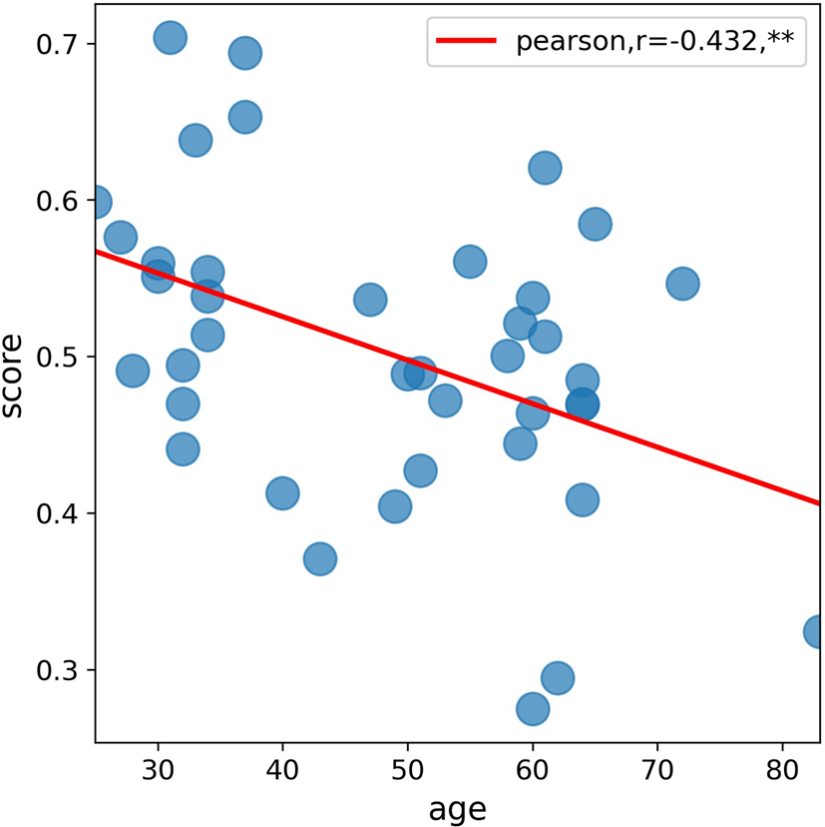
The relationship between the immune status scores and age in the independent validation dataset. The results indicated a significant negative correlation (r = − 0.432, **P < 0.01) between age and score

To assess the relationship between immune status score and age, we performed a Pearson correlation analysis. The analysis revealed a significant negative correlation between immune status score and age (r = − 0.432, *p* < 0.01), as shown in Fig. 6. This finding suggests that as age increases, the immune status score tends to decrease.

Overall, the results from the independent validation dataset support the validity and generalizability of our model in assessing immune status.

## Discussion

Our research has successfully developed a model for assessing immune status by processing, clustering, and conducting correlation analysis on the complete blood count (CBC) data of healthy adult individuals. By processing and analyzing the data from 16,715 healthy individuals, we discovered a correlation between immune status and age, and designed an assessment model that effectively monitors and evaluates individual immune health.

Firstly, we cleaned the CBC data by excluding individuals with mild inflammation to more accurately represent the immune status of healthy individuals. Subsequently, we normalized the CBC data of these 16,715 healthy individuals using a three-platform model and performed cluster analysis on their immune status using the EM-GMM algorithm. Through this series of data processing and analysis, we successfully divided these individuals into three groups: good, moderate, and poor immune status, and identified the correlation between immune status and age.

Secondly, we evaluated the correlation between various CBC indicators and the immune status of healthy individuals using the RF, LightGBM, and XGBoost models. We designed a comprehensive scoring system to assess individual immune status. We also visualized the trend of immune status changes with age using polynomial regression models and validated the effectiveness and universality of the model in assessing immune status.

Finally, we conducted practical tests and independent validations by monitoring the immune status of five healthy individuals for ten days and using an independent dataset for model verification. The experimental results demonstrated that the model has good stability and accuracy, effectively assessing individual immune status, and exhibiting good applicability with external validation data.

The contribution and innovation of this study lie in establishing an immune status assessment model using CBC data from 16,715 healthy individuals, and demonstrating the stability of the model through multiple independent tests, which is a large-scale study. In contrast, many earlier studies had smaller sample sizes, which limited their universality and reliability. Previous studies have mostly focused on assessing the immune status of patients, with limited research on the immune status of healthy individuals, which has only allowed for qualitative comparisons. Additionally, the quantitative methods used in previous studies have been too rudimentary, further highlighting the lack of comprehensive research in evaluating the immune status of healthy individuals. Our model is more scientific and actionable, facilitating early detection of health issues and providing important reference information for clinical medicine and public health.

In conclusion, the proposed immunological status assessment model in this study demonstrates significant potential for application in the field of immune health. However, considering the primary focus on cellular-level factors in this research, future investigations should further consider molecular-level factors such as TCR, BCR. that influence immune status, aiming to enhance and optimize the assessment model. Additionally, long-term follow-up observations of patients with lower immune status scores are necessary to validate the stability and reliability of the model.

## Conclusions

In conclusion, our study successfully developed the three-platform model for normalizing CBC data of healthy individuals. Through the use of advanced clustering and machine learning algorithms, we constructed an immune status evaluation model that allows for the assessment of an individual’s immune status by comparing their immunity score to age-specific reference values. Our findings highlighted the detrimental impact of insufficient sleep on immune status, as evidenced by lower immunity scores in individuals intentionally disrupting their immune status by staying up late. This evaluation method holds promise as an early warning system for disease risks, including susceptibility to COVID-19 infection. Our research underscores the potential of this model in assessing immune status and identifying influential factors such as sleep disruption and age. Further investigation is warranted to delve into the underlying mechanisms and implications of these findings.

### Supplementary Information

**Additional file 1**. Supplementary figures.

**Additional file 2**. Supplementary tables.

## Data Availability

The data and code are available online at https://github.com/zhangbeibei-min/Immune-Status-Assessment.git.

## Abbreviations

CBC: Complete blood counts
EM-GMM: A Gaussian mixture model was optimized with expectation–maximization
RF: Random forest
LightGBM: Light gradient boosting machine
XGBoost: Extreme gradient boosting
PCT: Procalcitonin
WBC: White blood cell count
LYMPH: Lymphocyte count
NEUT: Neutrophil count
MONO: Monocyte count
EO: Eosinophil count
BASO: Basophil count
LYMPH%: Lymphocyte percentage
NEUT%: Neutrophil percentage
MONO%: Monocyte percentage
EO%: Eosinophil percentage
BASO%: Basophil percentage
NLR: Neutrophil to lymphocyte ratio
MLR: Monocyte to lymphocyte ratio
ELR: Eosinophil to lymphocyte ratio
BLR: Basophils to lymphocytes ratio
