# Supplementary material for: A score-based method of immune status evaluation for healthy individuals with complete blood cell counts
Source: BMC Bioinformatics. 2023 Dec 11;24:467. doi: 10.1186/s12859-023-05603-7 (PMC10714576; doi:10.1186/s12859-023-05603-7)
Supplement: Supplementary file 1 — Additional file 1. Supplementary figures. [file 12859_2023_5603_MOESM1_ESM.doc]

**Supplementary Figures**


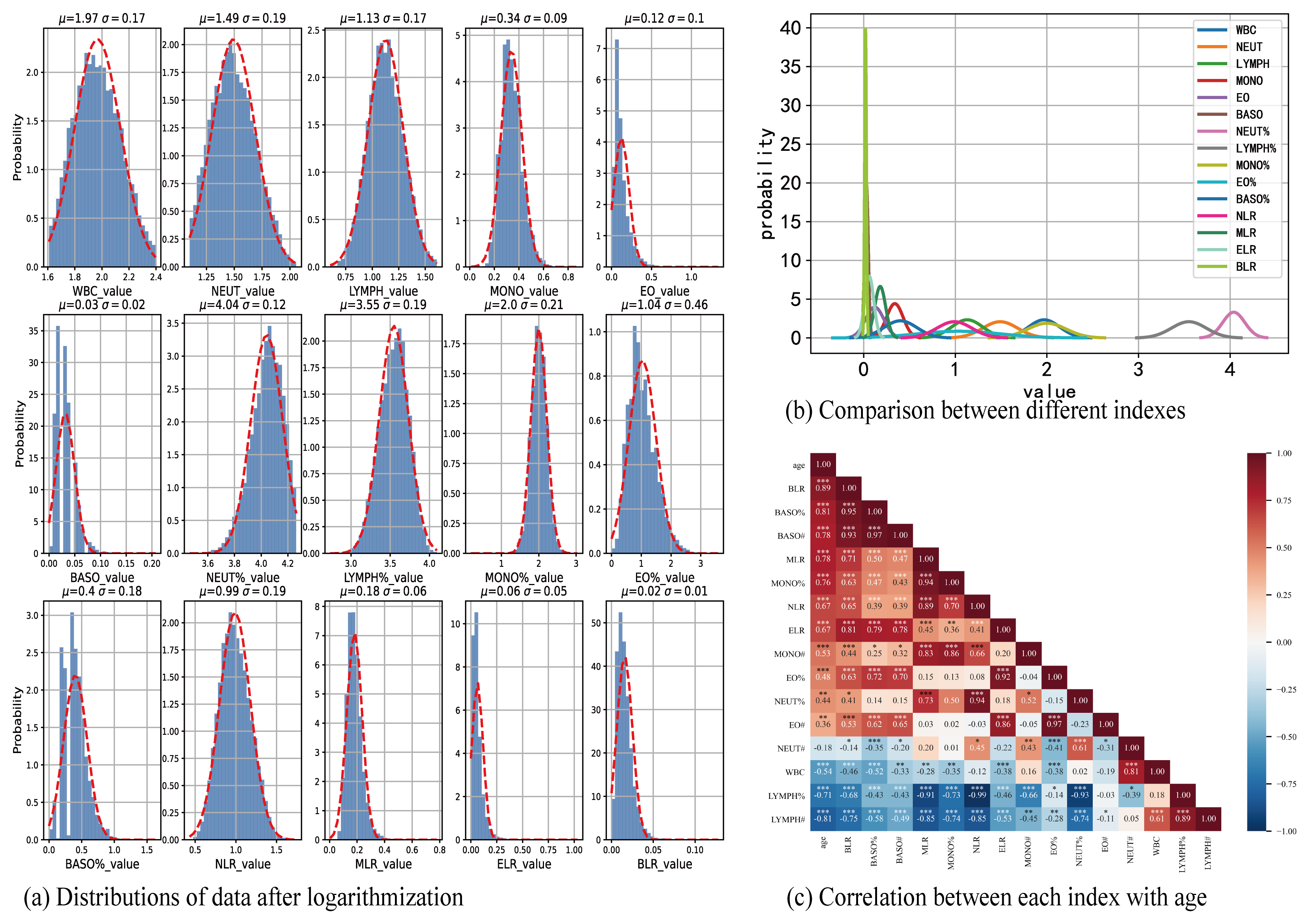


**Figure S1**. Profile of the complete blood count data. (a) Distributions of data after logarithmization. (b) Comparison between different indexes. (c) Correlation between each index with age. (red: positive, blue: negative).


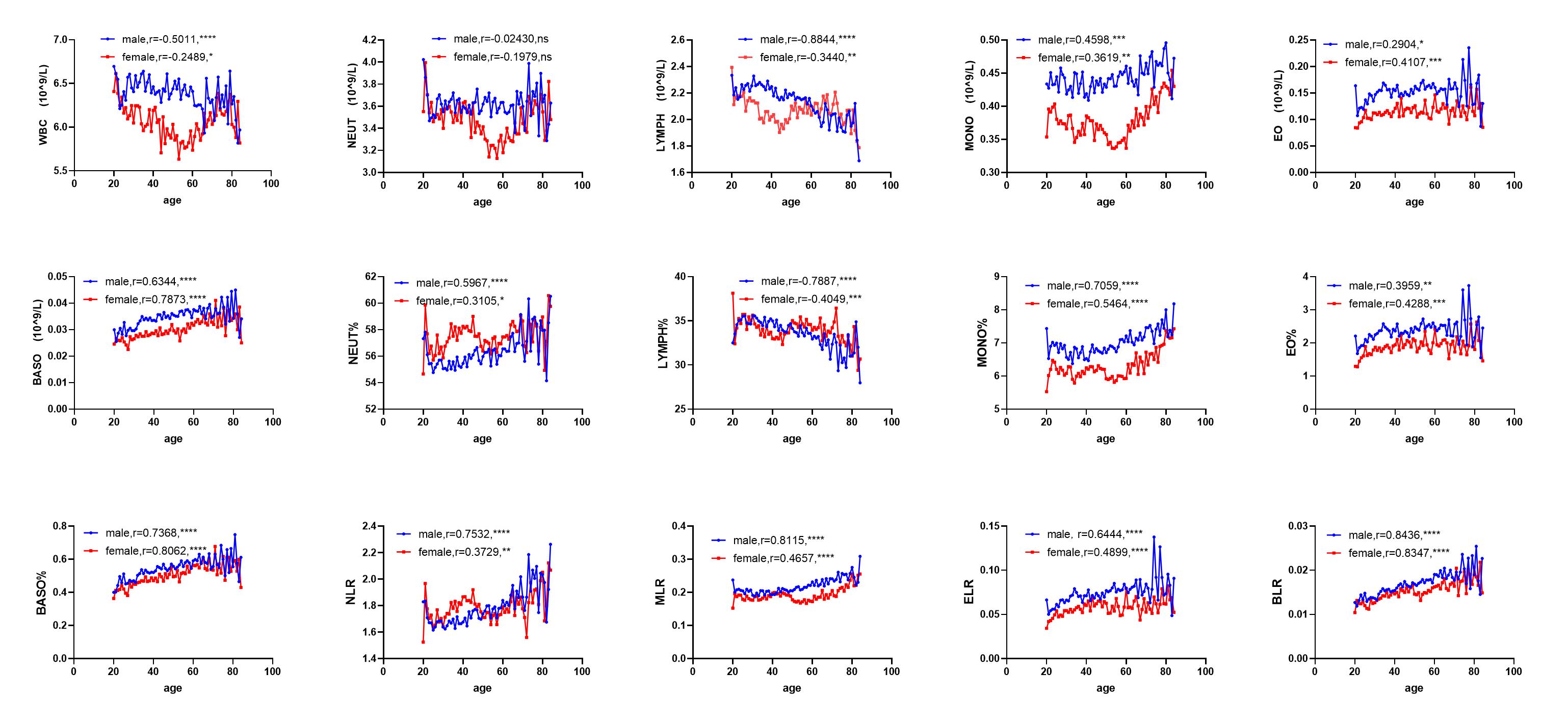


**Figure S2**. The trend of different gender indicators with age


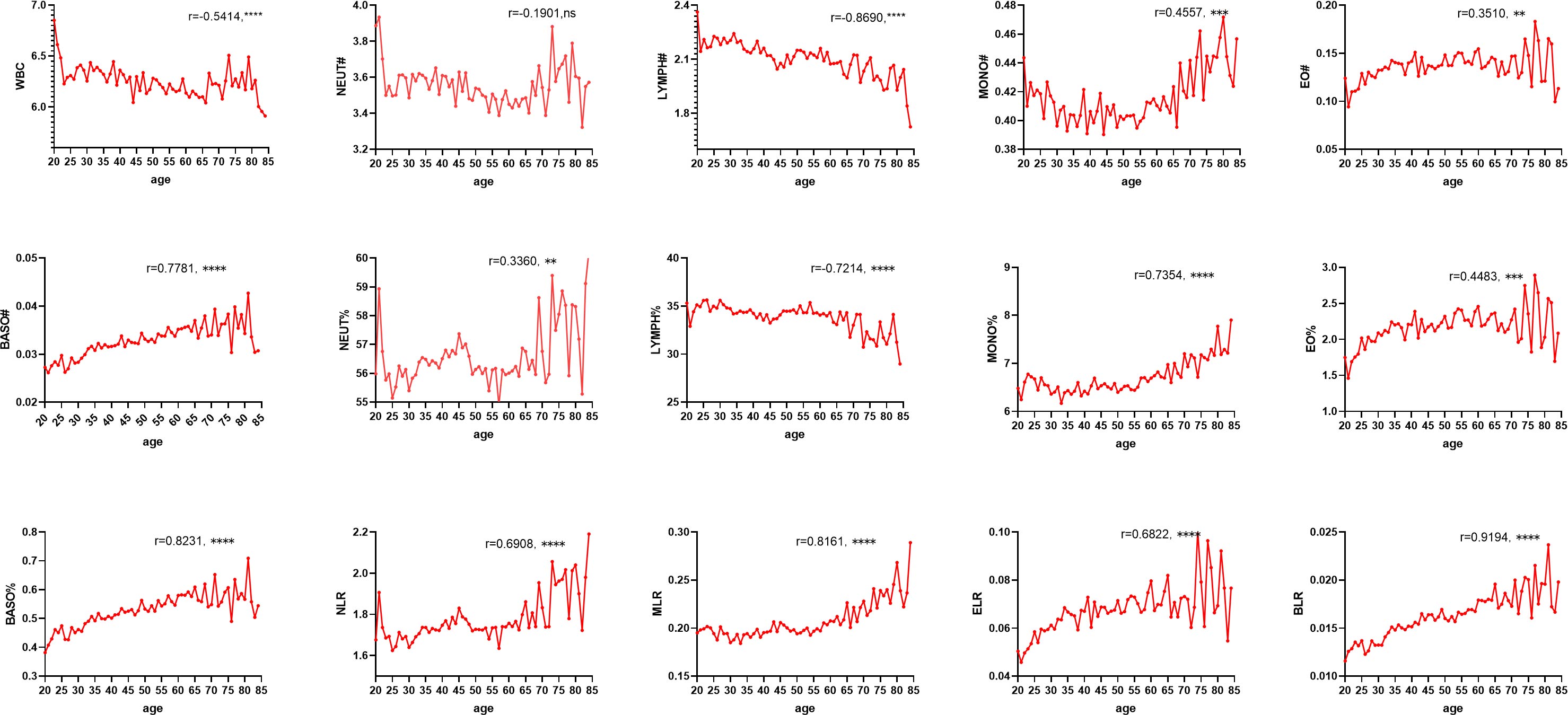


**Figure S3**. The trend of each index along with age


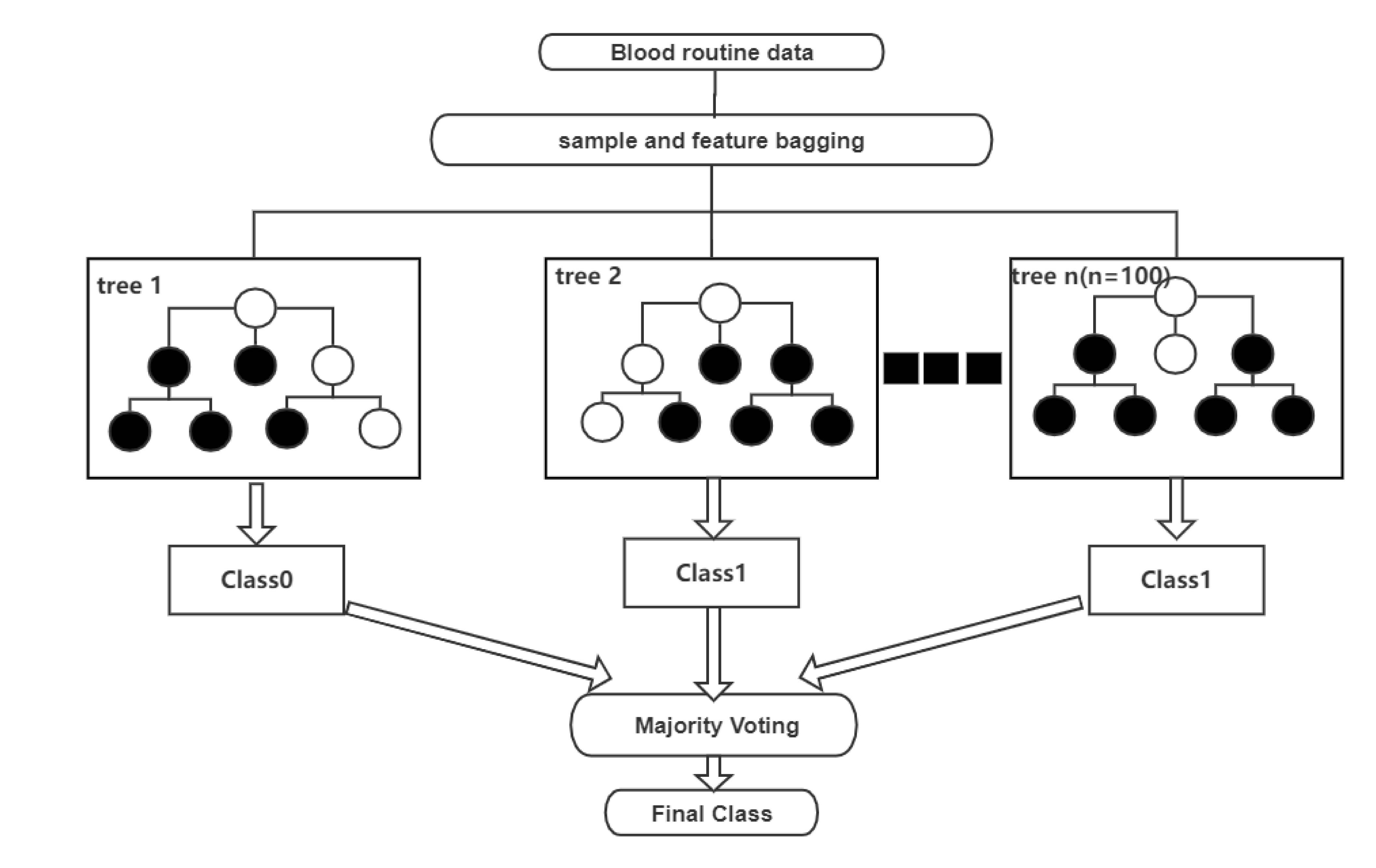


**Figure S4**. Random forest for evaluating the correlation of each CBC index with immune status


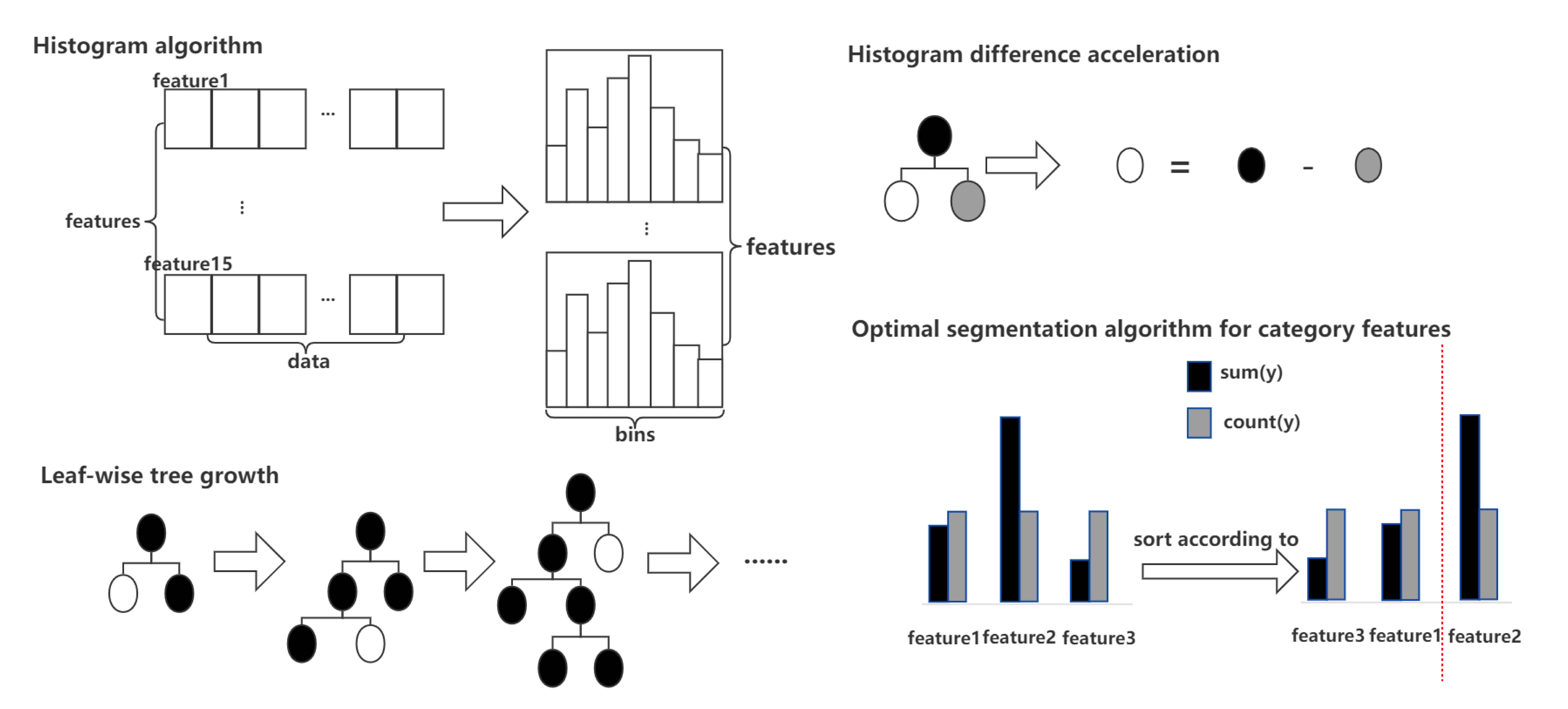


**Figure S5**. LightGBM for evaluating the correlation of each CBC index with immune status.


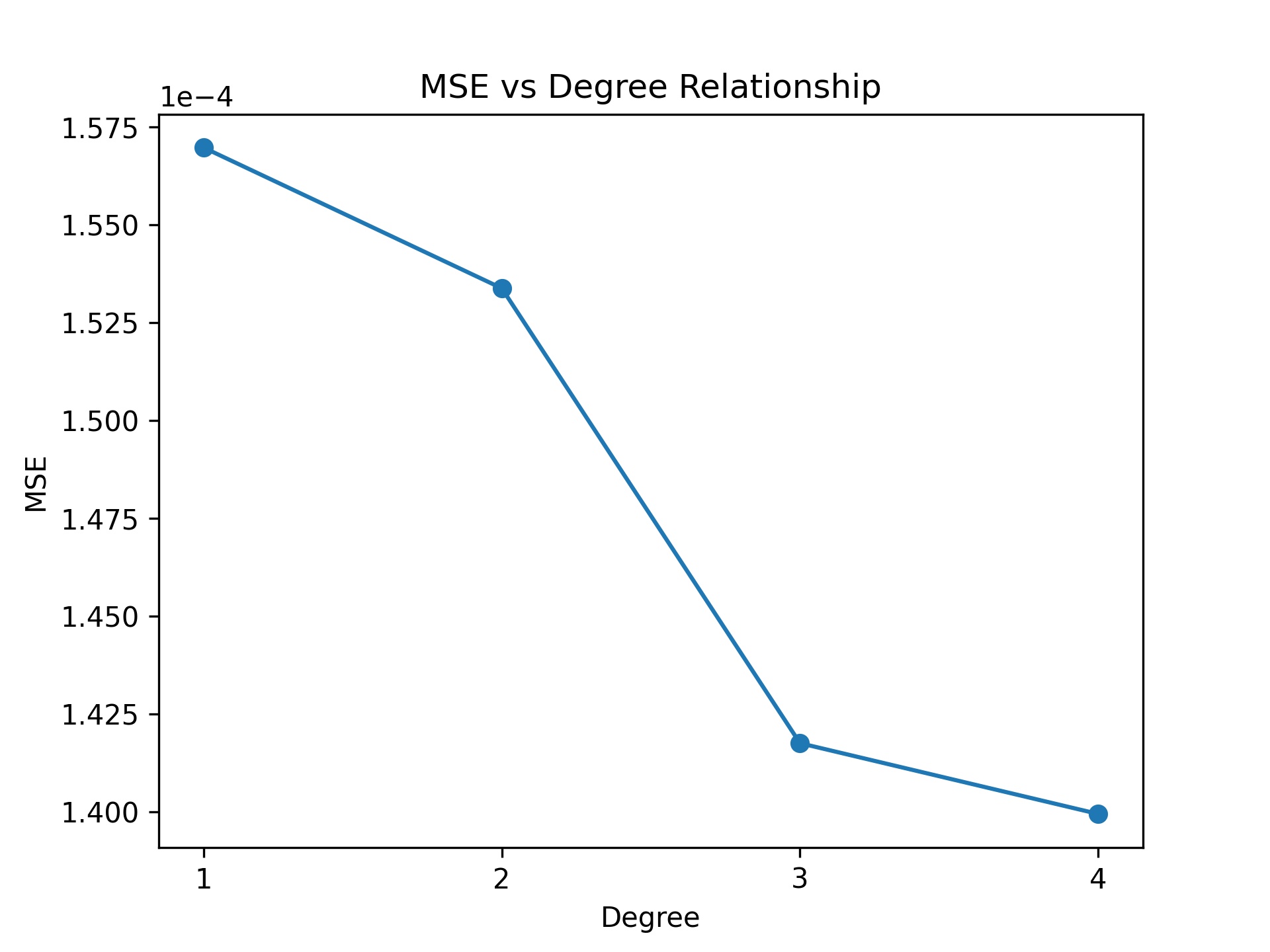


**Figure S6**. Variation of MSE with polynomial degree.


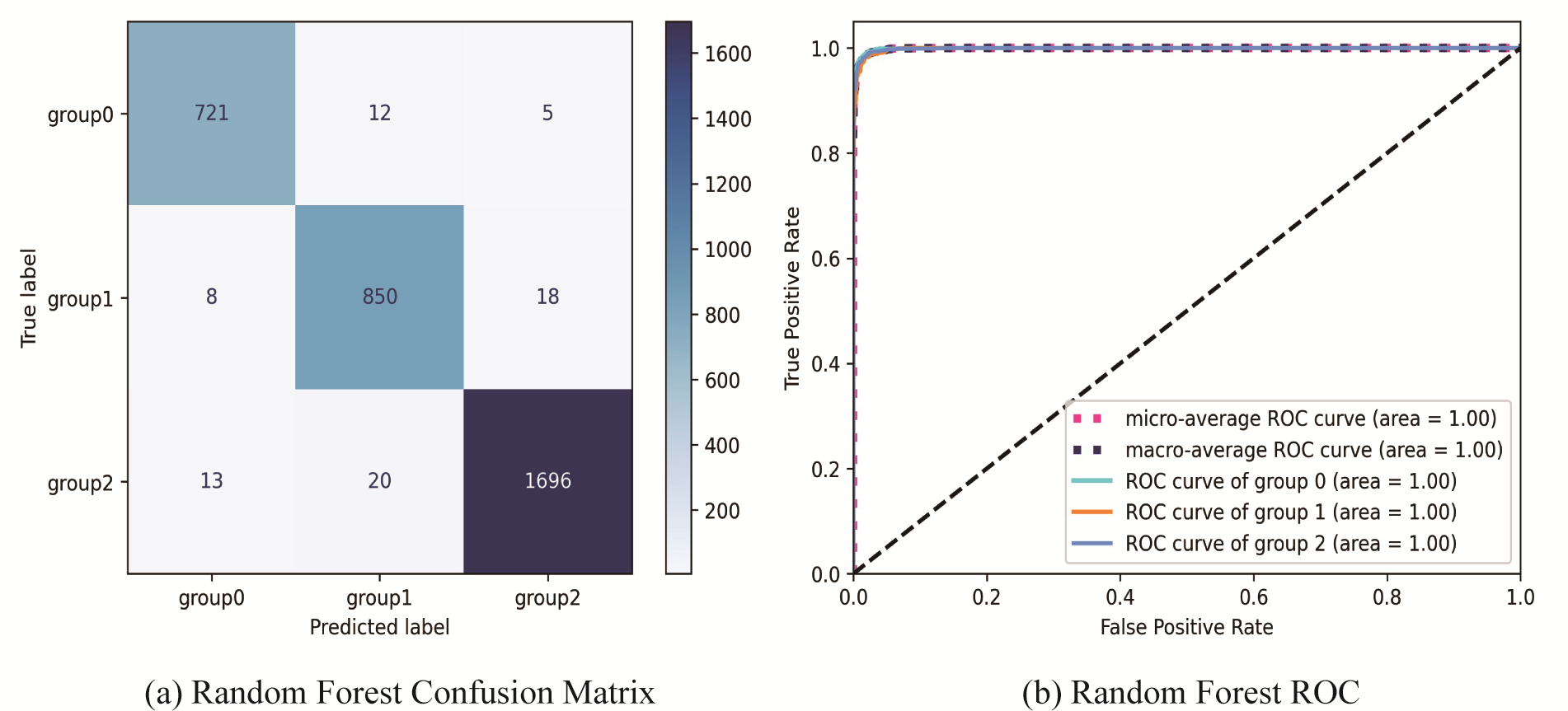


**Figure S7**. Test results of Random Forest. (a) Rand Forest Confusion Matrix. (b) Random Forest ROC.


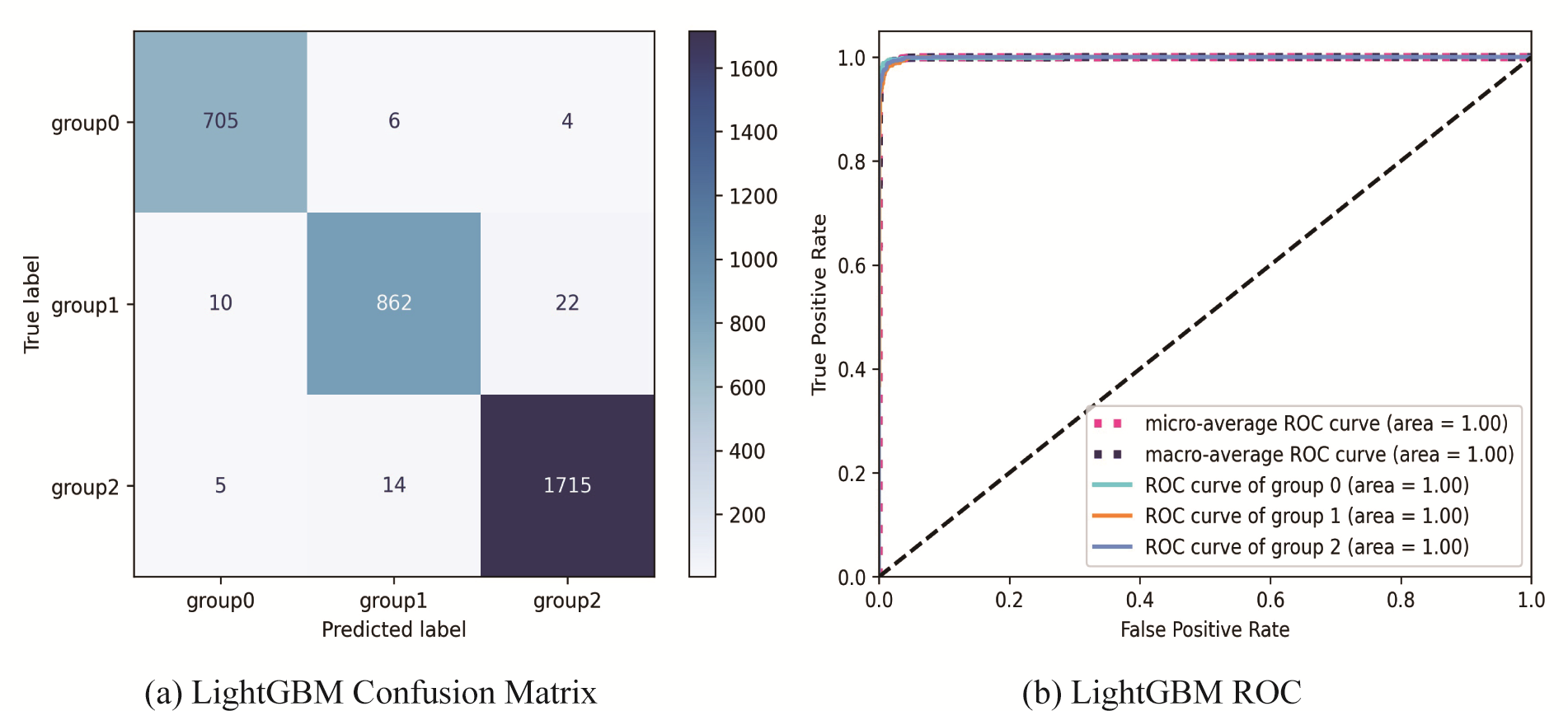


**Figure S8**. Test results of LightGBM. (a) LightGBM Confusion Matrix. (b) LightGBM ROC


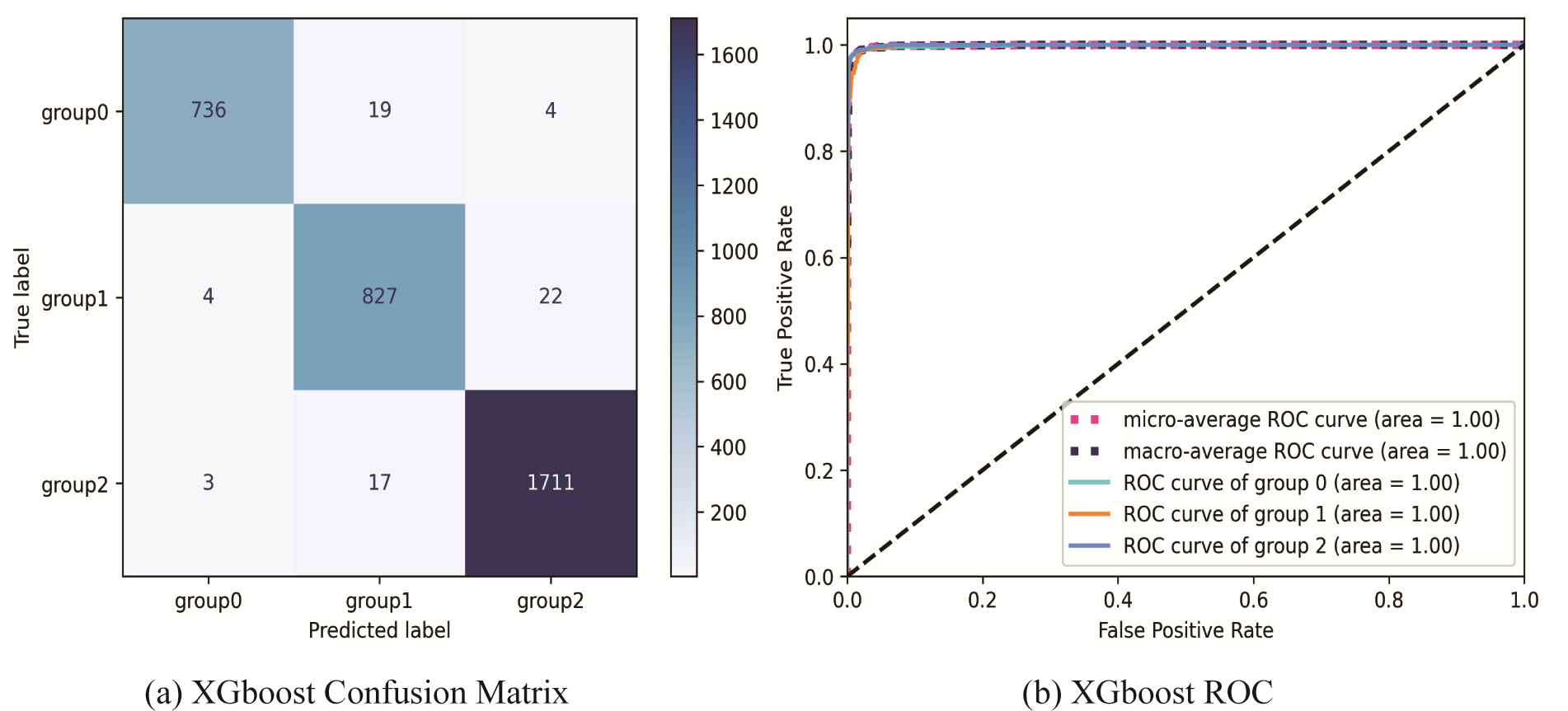


**Figure S9**. Test results of XGboost. (a) XGboost Confusion Matrix. (b) XGboost ROC


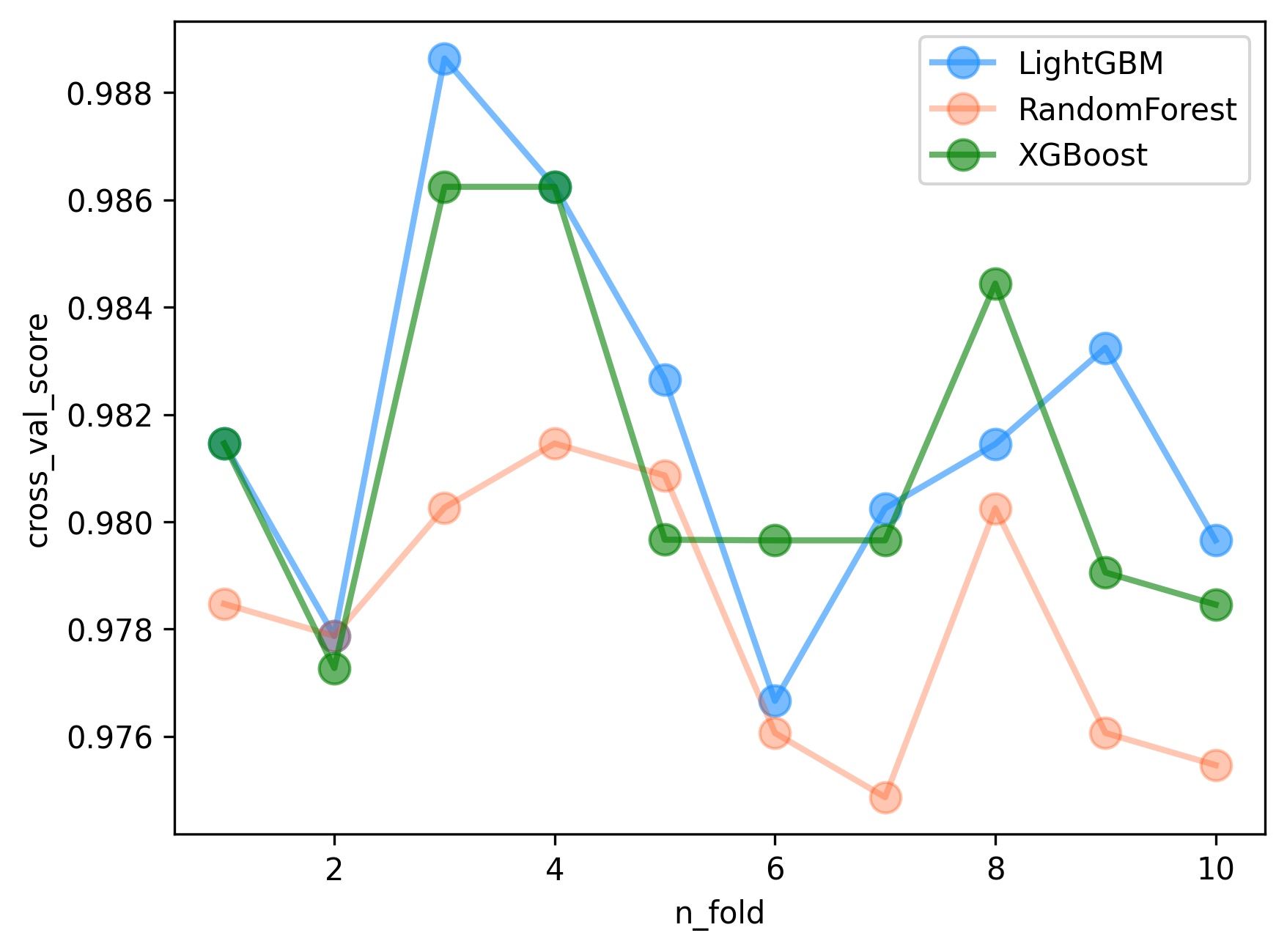


**Figure S10**. Test results of RandomForest, LightGBM and XGboost.
