# Supplementary material for: A score-based method of immune status evaluation for healthy individuals with complete blood cell counts
Source: BMC Bioinformatics. 2023 Dec 11;24:467. doi: 10.1186/s12859-023-05603-7 (PMC10714576; doi:10.1186/s12859-023-05603-7)
Supplement: Supplementary file 2 — Additional file 2. Supplementary tables. [file 12859_2023_5603_MOESM2_ESM.doc]

**Supplementary Table**

**Table S1** Reference range of blood routine data

| **parameter** | **Raw Data（10^9/L）** | | **Logarithm processing of Data** | |
| --- | --- | --- | --- | --- |
|  | Mean | SD | Mean | SD |
| **WBC** | 6.25 | 1.58 | 1.97 | 0.03 |
| **NEUT** | 3.54 | 0.82 | 1.49 | 0.04 |
| **LYMPH** | 2.13 | 0.28 | 1.13 | 0.03 |
| **MONO** | 0.41 | 0.02 | 0.34 | 0.01 |
| **EO** | 0.14 | 0.02 | 0.12 | 0.01 |
| **BASO** | 0.03 | 0.0004 | 0.04 | 0.0003 |
| **NEUT（%）** | 56.3 | 44.60 | 4.04 | 0.01 |
| **LYMPH（%）** | 34.28 | 41.77 | 3.55 | 0.03 |
| **MONO（%）** | 6.57 | 2.60 | 2.00 | 0.04 |
| **EO（%）** | 2.17 | 3.45 | 1.04 | 0.21 |
| **BASO（%）** | 0.52 | 0.09 | 0.40 | 0.03 |
| **NLR** | 1.74 | 0.29 | 0.99 | 0.04 |
| **MLR** | 0.20 | 0.01 | 0.18 | 0.003 |
| **ELR** | 0.07 | 0.004 | 0.06 | 0.003 |
| **BLR** | 0.02 | 9.32E-05 | 0.02 | 8.89E-05 |

**Table S2** The complete blood counts of Person1

| **Person1(healthy, 27 years, female)** | | | | | |
| --- | --- | --- | --- | --- | --- |
| **Date** | **Day1** | **Day3** | **Day5** | **Day7** | **Day9** |
| **WBC** | 5.17 | 5.52 | 7.22 | 6.24 | 7.01 |
| **NEUT** | 1.90 | 2.14 | 3.87 | 2.43 | 3.6 |
| **LYMPH** | 2.78 | 2.87 | 2.74 | 3.24 | 2.81 |
| **MONO** | 0.36 | 0.39 | 0.45 | 0.45 | 0.49 |
| **EO** | 0.07 | 0.06 | 0.09 | 0.06 | 0.05 |
| **BASO** | 0.05 | 0.05 | 0.06 | 0.05 | 0.04 |
| **NEUT%** | 36.62 | 38.70 | 53.70 | 38.9 | 51.3 |
| **LYMPH%** | 53.81 | 52.01 | 38.00 | 51.91 | 40.1 |
| **MONO%** | 7.00 | 7.10 | 6.20 | 7.20 | 7.00 |
| **EO%** | 1.40 | 1.10 | 1.20 | 1.00 | 0.70 |
| **BASO%** | 1.00 | 0.90 | 0.80 | 0.80 | 0.60 |
| **NLR** | 0.68 | 0.75 | 1.41 | 0.75 | 1.28 |
| **MLR** | 0.13 | 0.14 | 0.16 | 0.14 | 0.17 |
| **ELR** | 0.03 | 0.02 | 0.03 | 0.02 | 0.02 |
| **BLR** | 0.02 | 0.02 | 0.02 | 0.02 | 0.01 |

**Table S3.** The complete blood counts of Person2

| **Person2(healthy, 23 years, male)** | | | | | |
| --- | --- | --- | --- | --- | --- |
| **Date** | **Day1** | **Day3** | **Day5** | **Day7** | **Day9** |
| **WBC** | 6.63 | 8.53 | 5.94 | 6.40 | 6.65 |
| **NEUT** | 3.06 | 5.03 | 2.82 | 3.31 | 3.18 |
| **LYMPH** | 2.86 | 2.67 | 2.56 | 2.51 | 2.70 |
| **MONO** | 0.52 | 0.66 | 0.40 | 0.44 | 0.60 |
| **EO** | 0.15 | 0.12 | 0.12 | 0.10 | 0.13 |
| **BASO** | 0.03 | 0.03 | 0.02 | 0.03 | 0.03 |
| **NEUT%** | 46.10 | 59.00 | 47.60 | 51.6 | 47.70 |
| **LYMPH%** | 42.10 | 31.30 | 43.10 | 39.20 | 40.60 |
| **MONO%** | 7.80 | 7.70 | 6.70 | 6.90 | 9.00 |
| **EO%** | 2.30 | 1.40 | 2.00 | 1.60 | 2.00 |
| **BASO%** | 0.50 | 0.40 | 0.30 | 0.50 | 0.50 |
| **NLR** | 1.07 | 1.88 | 1.10 | 1.32 | 1.18 |
| **MLR** | 0.18 | 0.25 | 0.16 | 0.18 | 0.22 |
| **ELR** | 0.05 | 0.04 | 0.05 | 0.04 | 0.05 |
| **BLR** | 0.01 | 0.01 | 0.01 | 0.01 | 0.01 |

**Table S4** The complete blood counts of Person3

| **Person3(healthy, 26 years, female)** | | | | | |
| --- | --- | --- | --- | --- | --- |
| **Date** | **Day1** | **Day3** | **Day5** | **Day7** | **Day9** |
| **WBC** | 5.24 | 4.44 | 4.78 | 3.50 | 3.90 |
| **NEUT** | 2.63 | 2.00 | 2.82 | 1.51 | 1.89 |
| **LYMPH** | 2.19 | 2.02 | 1.64 | 1.73 | 1.69 |
| **MONO** | 0.38 | 0.36 | 0.27 | 0.23 | 0.26 |
| **EO** | 0.01 | 0.03 | 0.03 | 0.02 | 0.03 |
| **BASO** | 0.02 | 0.02 | 0.01 | 0.01 | 0.02 |
| **NEUT%** | 50.10 | 45.00 | 59.10 | 43.10 | 48.40 |
| **LYMPH%** | 41.80 | 45.50 | 34.30 | 49.40 | 43.30 |
| **MONO%** | 7.30 | 8.10 | 5.60 | 6.60 | 6.70 |
| **EO%** | 0.20 | 0.70 | 0.60 | 0.60 | 0.80 |
| **BASO%** | 0.40 | 0.50 | 0.20 | 0.30 | 0.50 |
| **NLR** | 1.20 | 0.99 | 1.72 | 0.87 | 1.12 |
| **MLR** | 0.17 | 0.18 | 0.16 | 0.13 | 0.15 |
| **ELR** | 0.00 | 0.01 | 0.02 | 0.01 | 0.02 |
| **BLR** | 0.01 | 0.01 | 0.01 | 0.01 | 0.01 |

**Table S5** The complete blood counts of Person4

| **Person4(healthy, 29 years, male)** | | | | | |
| --- | --- | --- | --- | --- | --- |
| **Date** | **Day1** | **Day3** | **Day5** | **Day7** | **Day9** |
| **WBC** | 7.13 | 8.87 | 8.64 | 9.00 | 7.71 |
| **NEUT** | 2.68 | 3.35 | 3.86 | 4.26 | 3.64 |
| **LYMPH** | 3.27 | 4.12 | 3.36 | 3.29 | 2.72 |
| **MONO** | 0.62 | 0.81 | 0.70 | 0.80 | 0.70 |
| **EO** | 0.50 | 0.53 | 0.65 | 0.58 | 0.58 |
| **BASO** | 0.05 | 0.04 | 0.06 | 0.05 | 0.06 |
| **NEUT%** | 37.60 | 37.84 | 44.70 | 47.30 | 47.20 |
| **LYMPH%** | 45.90 | 46.44 | 38.90 | 36.60 | 35.30 |
| **MONO%** | 8.70 | 9.14 | 8.10 | 8.90 | 9.10 |
| **EO%** | 7.01 | 6.00 | 7.51 | 6.41 | 7.51 |
| **BASO%** | 0.70 | 0.50 | 0.70 | 0.60 | 0.80 |
| **NLR** | 0.82 | 0.81 | 1.15 | 1.29 | 1.34 |
| **MLR** | 0.19 | 0.20 | 0.21 | 0.24 | 0.26 |
| **ELR** | 0.15 | 0.13 | 0.19 | 0.18 | 0.21 |
| **BLR** | 0.02 | 0.01 | 0.02 | 0.02 | 0.02 |

**Table S6** The complete blood counts of Person5

| **Person5(healthy, 23 years, male)** | | | | | |
| --- | --- | --- | --- | --- | --- |
| **Date** | **Day1** | **Day3** | **Day5** | **Day7** | **Day9** |
| **WBC** | 5.28 | 6.19 | 5.72 | 4.75 | 5.93 |
| **NEUT** | 2.49 | 3.41 | 3.12 | 2.68 | 3.16 |
| **LYMPH** | 2.22 | 2.12 | 2.01 | 1.61 | 2.17 |
| **MONO** | 0.42 | 0.48 | 0.42 | 0.33 | 0.45 |
| **EO** | 0.10 | 0.11 | 0.11 | 0.09 | 0.10 |
| **BASO** | 0.04 | 0.06 | 0.05 | 0.03 | 0.04 |
| **NEUT%** | 47.10 | 55.00 | 54.60 | 56.50 | 53.20 |
| **LYMPH%** | 42.00 | 34.20 | 35.10 | 33.90 | 36.60 |
| **MONO%** | 8.00 | 7.80 | 7.30 | 6.90 | 7.60 |
| **EO%** | 1.90 | 1.80 | 1.90 | 1.90 | 1.70 |
| **BASO%** | 0.80 | 1.00 | 0.90 | 0.60 | 0.70 |
| **NLR** | 1.12 | 1.61 | 1.55 | 1.66 | 1.46 |
| **MLR** | 0.19 | 0.23 | 0.21 | 0.20 | 0.21 |
| **ELR** | 0.05 | 0.05 | 0.05 | 0.06 | 0.05 |
| **BLR** | 0.02 | 0.03 | 0.02 | 0.02 | 0.02 |
